# Supplementary material for: Assessing Microbial Activity and Rhizoremediation in Hydrocarbon and Heavy Metal-Impacted Soil
Source: Microorganisms. 2025 Apr 8;13(4):848. doi: 10.3390/microorganisms13040848 (PMC12029208; doi:10.3390/microorganisms13040848)
Supplement: Supplementary file 1 [file microorganisms-13-00848-s001.zip › microorganisms-3490519-supplementary.pdf]

Comparisons of Ryegrass 1 (Left) and Mustard (Right) root structures in the contaminated soil.

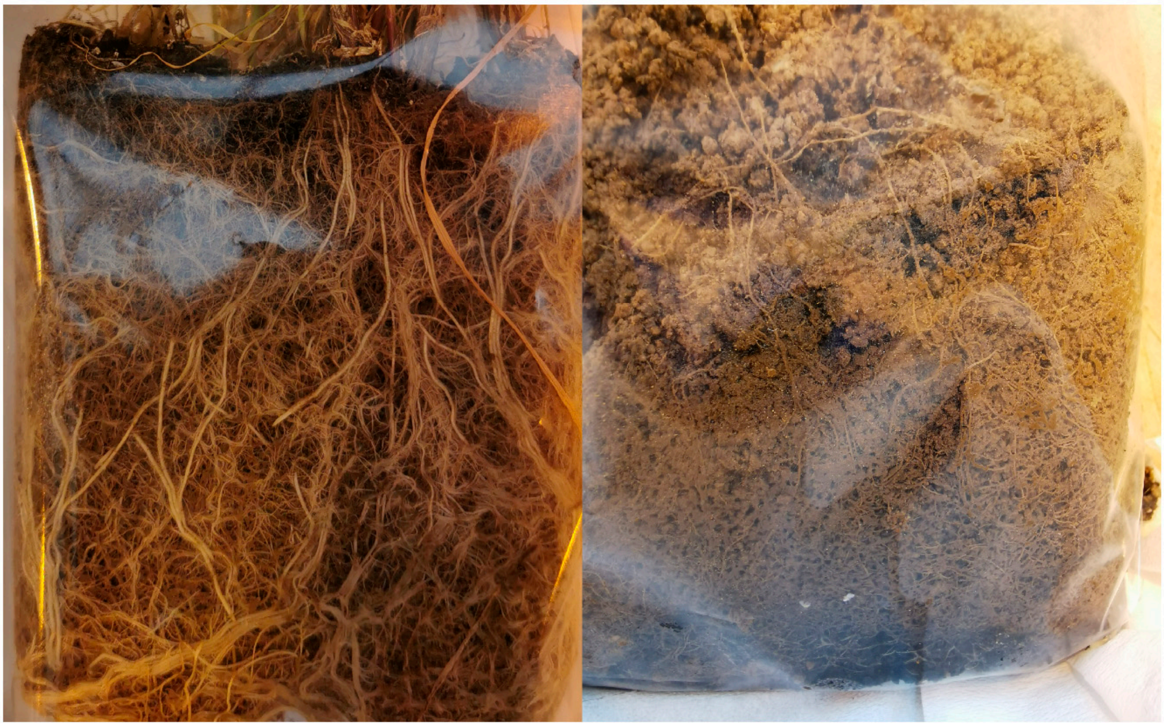

Figure S1: Is a photograph of ryegrass roots (abergain) vs mustard roots, illustrating the difference in root structure. Chicory roots were not visible this way due to most of the biomass centralised in the large taproot.

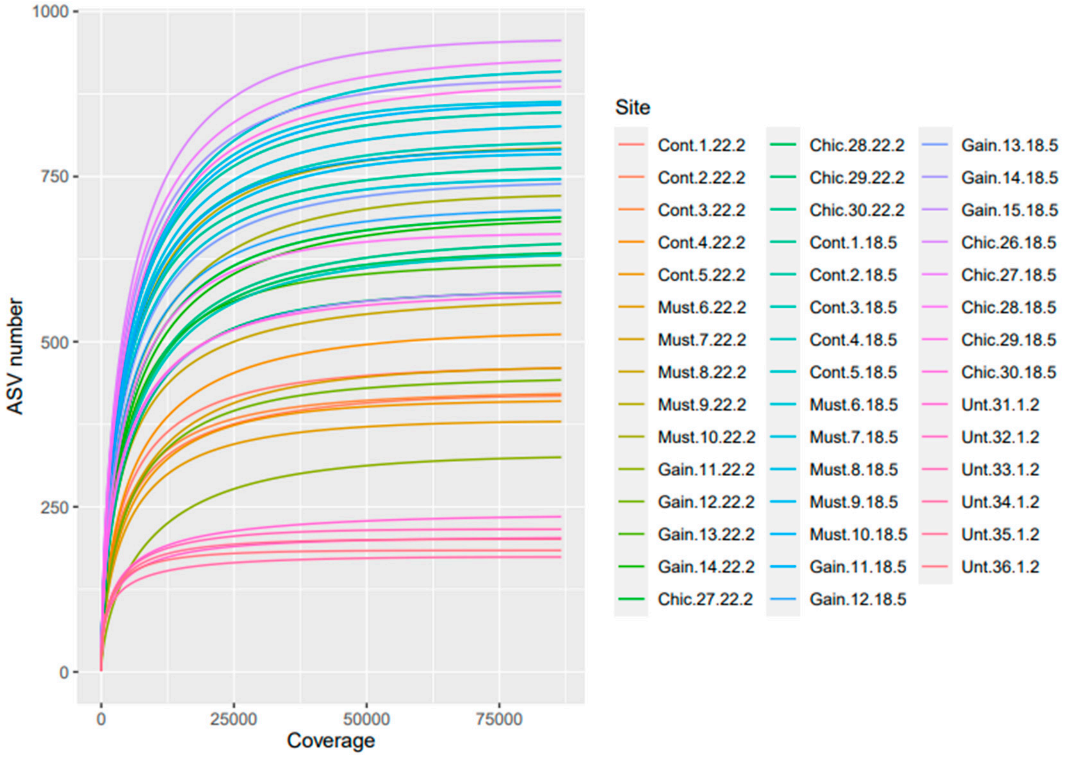

Figure S2 shows the rarefaction curve for bacteria prior to rarefaction with a sampling depth of 87,000 Raw tags after normalization to 95% of the minimum sampling depth (n = 5).

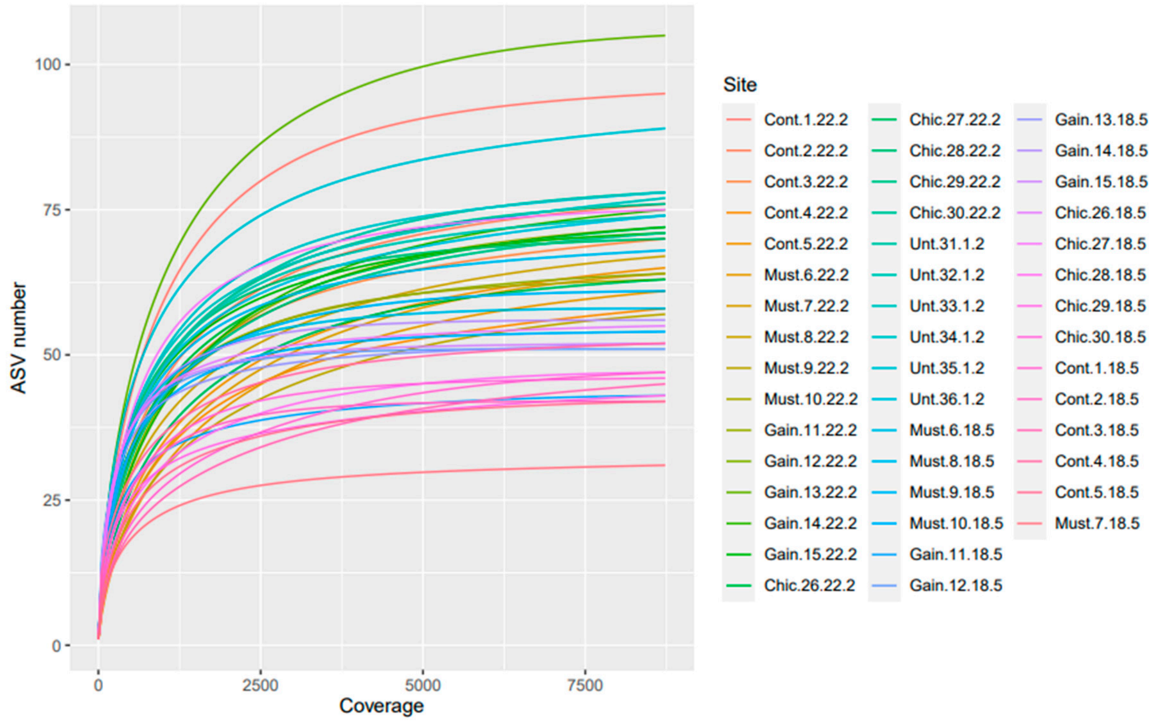

Figure S3: shows the rarefaction curve for fungi. The data was rarefied using R studio to simulate an even number of reads per sample. The rarefaction depth chose is 95% of the minimum sample depth in the dataset (n = 5).

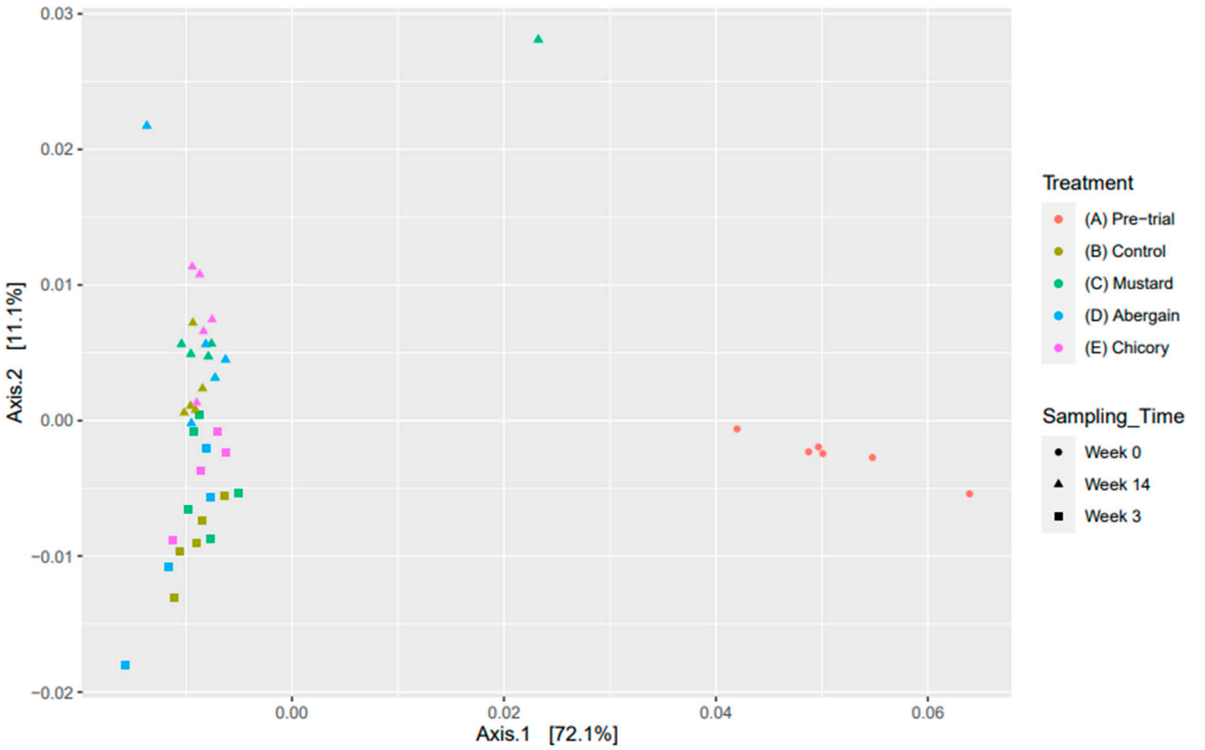

Figure S4 : Principal coordinate analysis of Weighted UniFrac for the Bacterial communities over the course of the 14 weeks. All replicates shown (n=5).

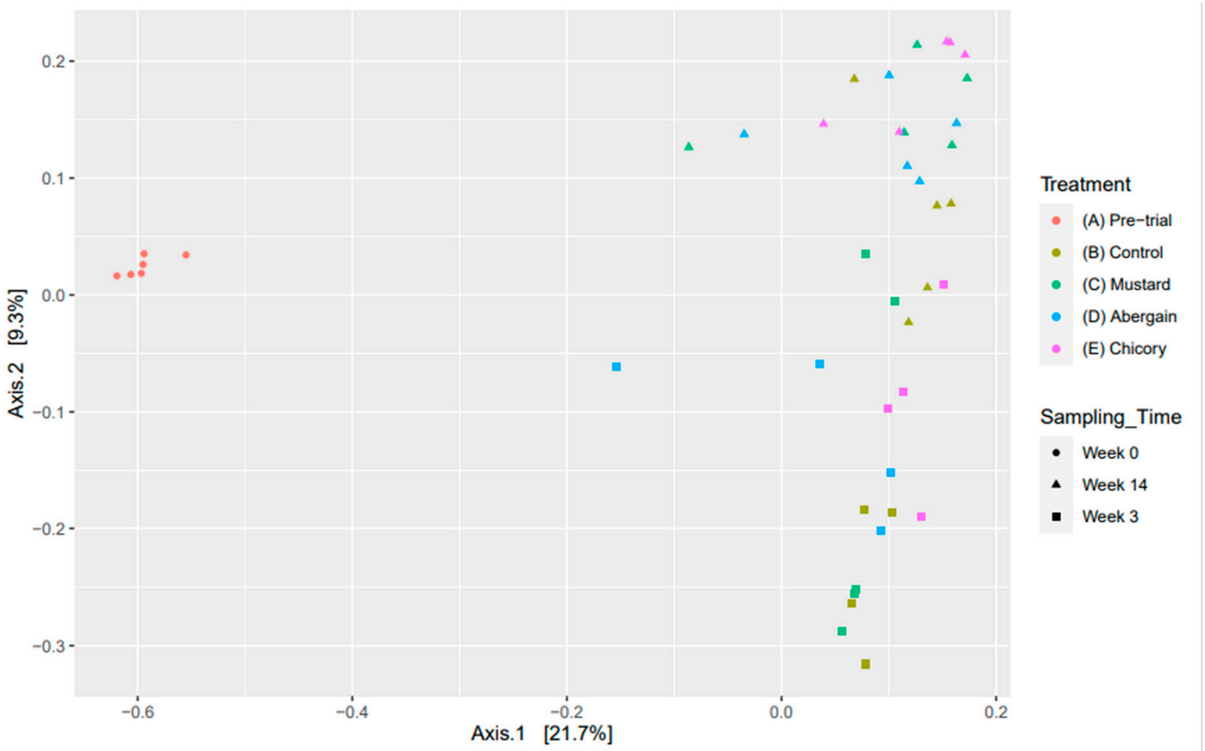

Figure S5: Principal coordinate analysis of Un-weighted UniFrac for the Bacterial communities over the course of the 14 weeks. All replicates shown (n=5).

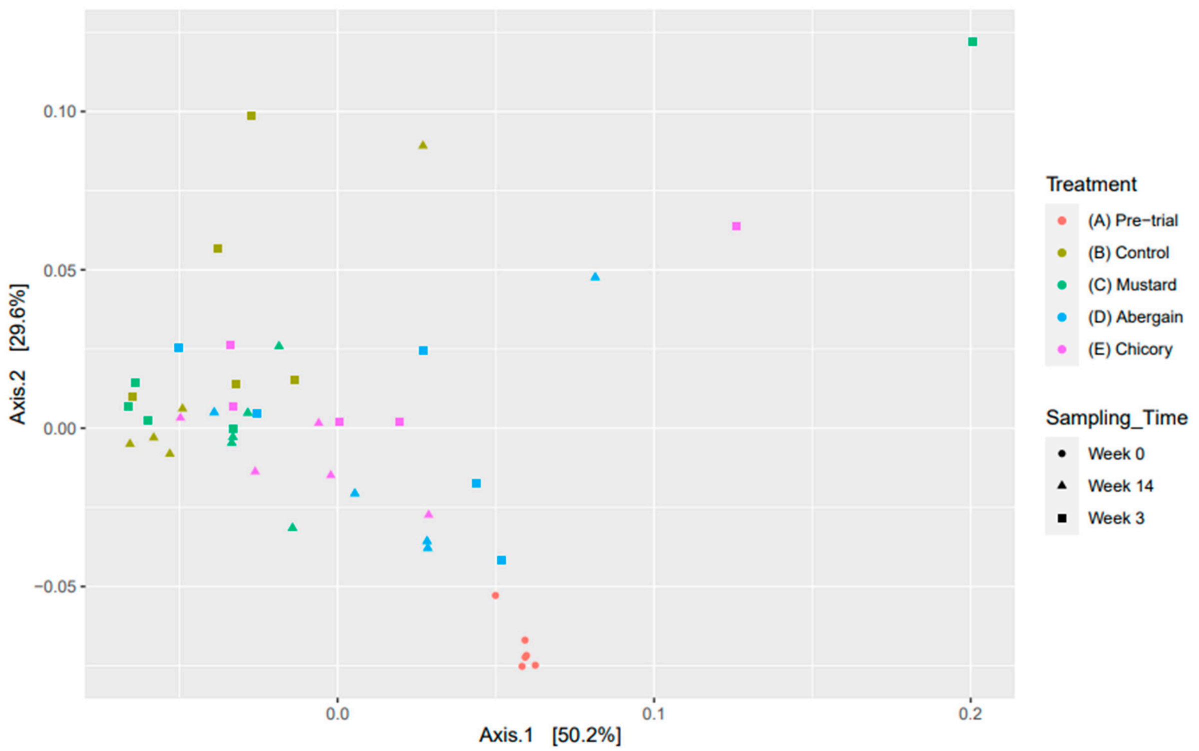

Figure S6: Principal coordinate analysis of Weighted UniFrac for the Fungi communities over the course of the 14 weeks. All replicates shown (n=5).

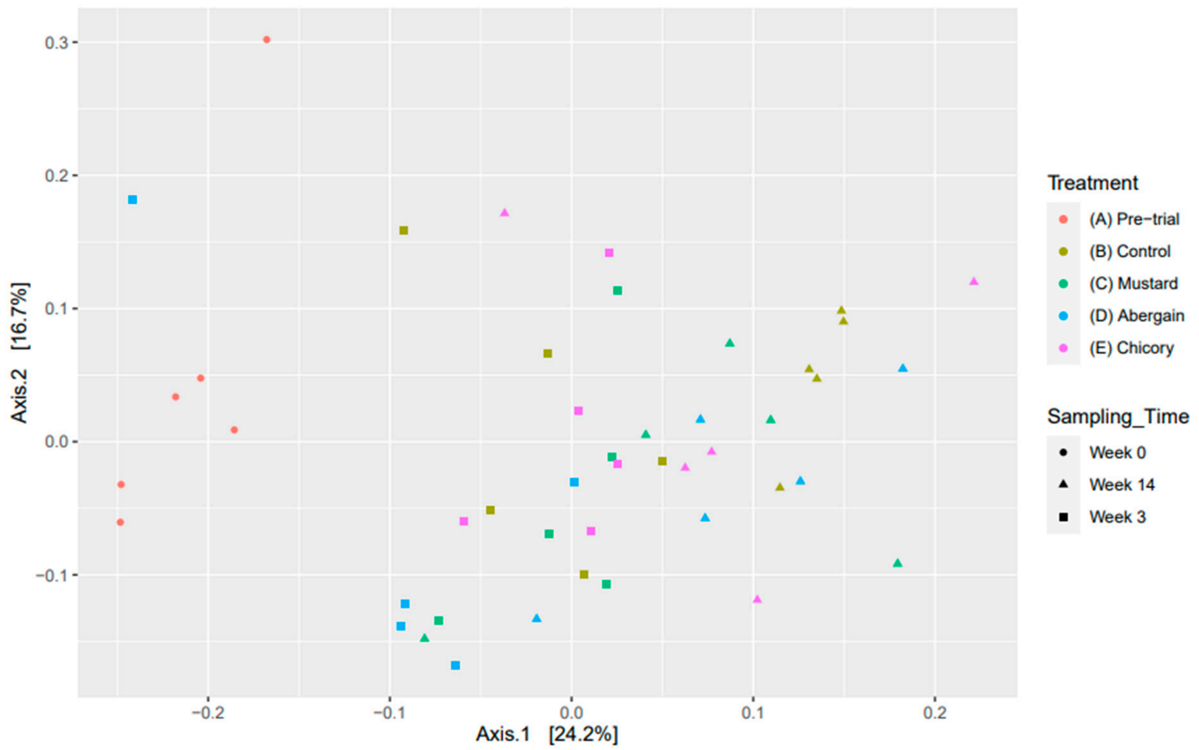

Figure S7: Principal coordinate analysis of Un-weighted UniFrac for the Fungi communities over the course of the 14 weeks. All replicates shown (n=5).

S1.1 Fungi Community Dynamics and Diversity During Phytoremediation

Fungi were sequenced at a sampling depth of 50,000 raw tags. To achieve consistent sequencing depth for all samples, the data was rarefied to a depth of 95% of the minimum sample depth in this data set, which resulted in a coverage of just under 9,000 raw tags. The rarefaction curves had plateaued at this point, suggesting adequate sampling depth for capturing the majority of species present (Figure S3). Information on observed ASVs, Chao1, Shannon, Simpsons inverse, weighted / unweighted unifrac and Bray-Curtis distances can be found in Table S4. Pretrial (Week 0) had the highest mean observed ASVs, this was not statistically significant compared to any treatments by week 3, but by week 14 all treatments had significantly decreased in observed ASVs ($P < 0.05$). Statistical significance of observed ASVs, Chao1, Simpsons Inverse and Shannon index can be found in Table S4.

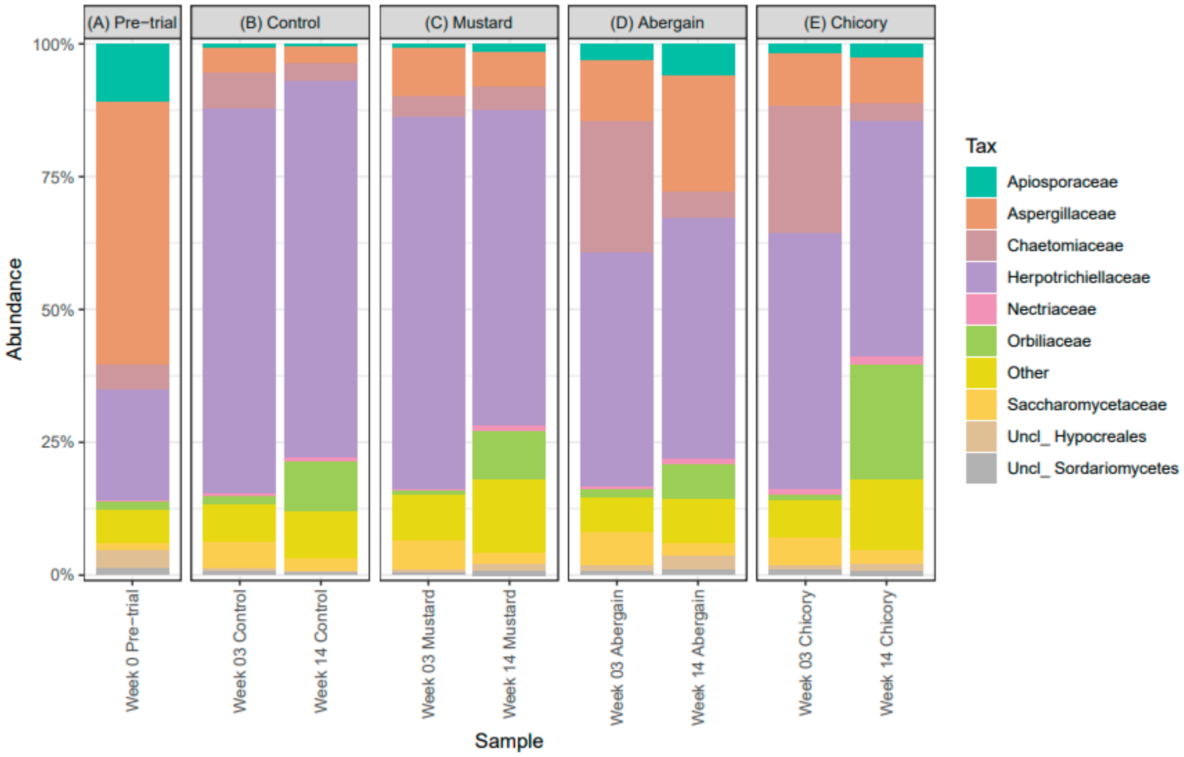

Figure S8: The major families of fungi found at each sampling time. Samples are grouped by treatment (top) and sampling date (bottom). (n = 5).

Figure S8 shows the top 10 fungal families found in each treatment. In the soil at the start of the experiment. The main families were *Aspergillaceae* (49%), *Herpotrichielaceae* (20.1%), *Apiosporaceae* (10.9%), *Chaetomiaceae* (4.5%) and *Orbiliaceae* (1%). By week 3 the following fungal families saw increases in relative abundances for all treatments *Herpotricielaceae* (Ranging: 44 – 72%) and *Chaetomiaceae* (Ranging: 3.8 – 24.6%). There was also a large decrease in *Aspergillaceae* (Ranging: 4.7 – 11.6%) in this time period. By week 14 there was an increase in *Orbiliaceae* (Ranging: 6.3 – 21.5%), a decrease in *Chaetomiaceae* (Ranging: 3.3 – 4.9%) and the other families remained relatively static.

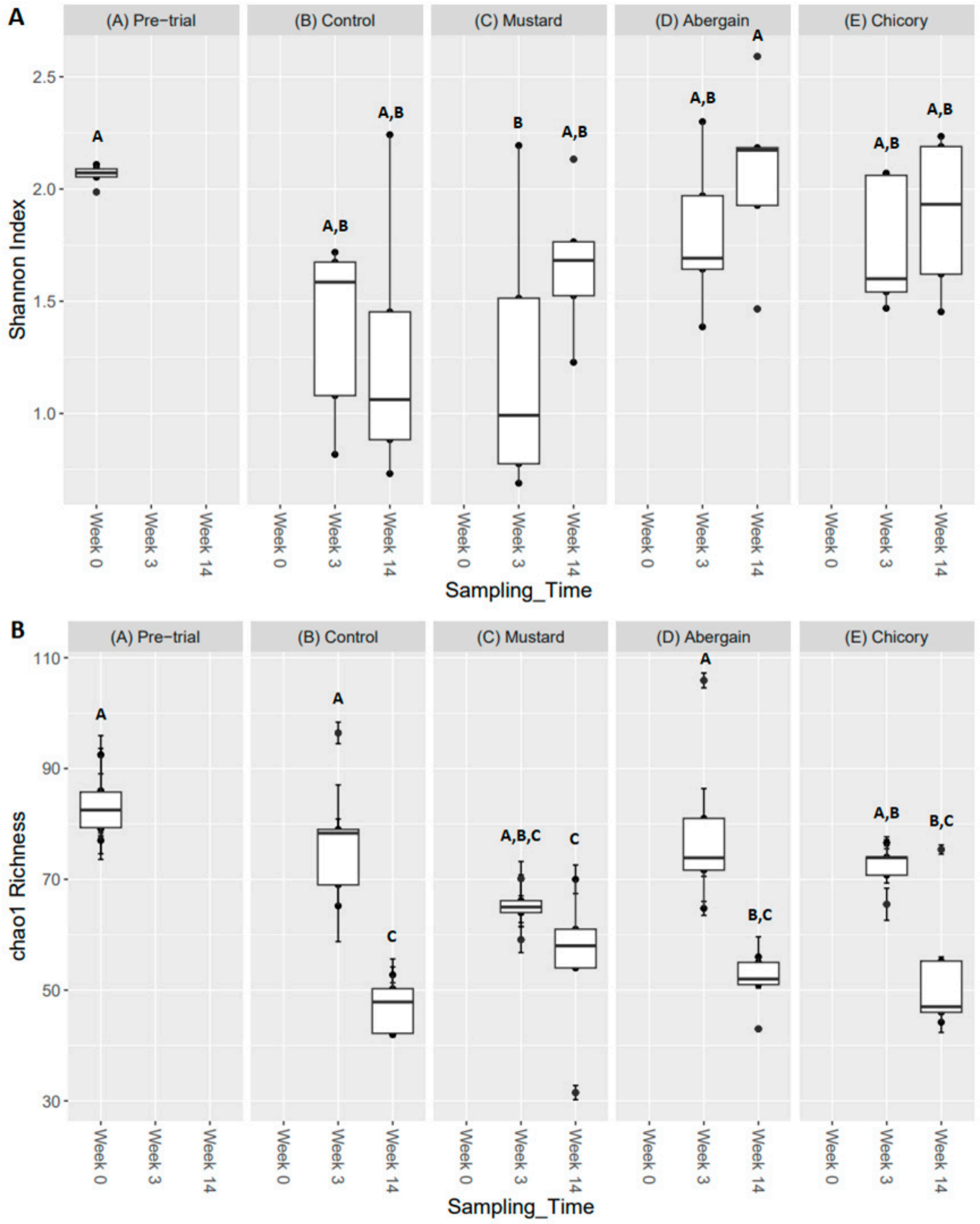

Figure S9: Fungal alpha diversity using the Shannon index (A) and Chao1 (B). Samples are grouped by treatment and all three sampling times are represented (n=5). Groups that are not statistically different share the same letter below the sampling date. Tukey was used to determine statistical significance for Shannon and chao1 (both normal distribution).

Figure S9 shows the fungal Shannon index (A) and Chao1 (B). For Shannon diversity the only statistically significant change from pre-trial was a decrease for Mustard at the week 14 sampling time. However, this was not significantly different from any other treatment except for Ryegrass (Abergain) at week 14. Chao1 richness found that all treatments at week 14 were significantly lower that the pre-

trial level, but there was no significant difference between the planted treatments at week 14. Simpsons inverse index, also showed that only the control and Mustard at week 14 had significantly changed. Figure S10 shows results for fungal beta diversity using Bray-Curtis distance. The analysis shows much more variation between and within treatments when compared with the bacterial data. Figure S5 shows the fungal beta diversity using weighted unifrac; although there is more precision between treatments and replicates compared to the Bray-Curtis distance, the data points are still not tightly clustered. This indicates that the fungal populations in samples are quite different in a direct comparison of species, but also not closely related on a phylogenetic scale.. There is a clear shift in fungal communities between weeks 0 and 3, however by week 14 fungal communities have not changed much.

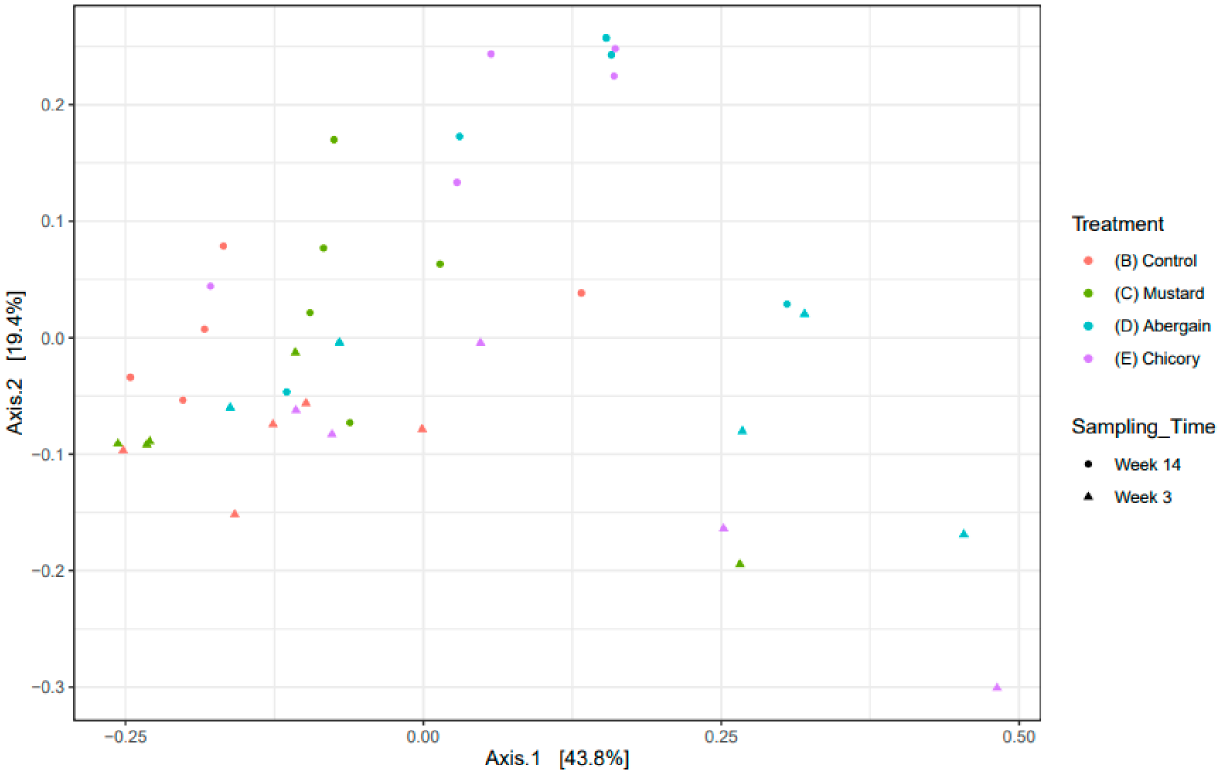

Figure S10: Principal coordinate analysis of the treatments using Bray-Curtis distances to visualise fungal beta diversity. All replicates are included (n=5). Pre-trial data points were removed to give better resolution for differences between treatments.

Figure S11 (A), (B) and (C) show the differential abundances at week 14 for the pre-trial versus the Control and the Control versus Mustard and Ryegrass respectively to genus level (also at week 14). Chicory is not included as there was no significant changes. In general, fungi were enriched in the plant treatments when compared to the control, except for *Arthrobtrys oligospora* which was depleted in the mustard treatment by a log2 fold change of -4. Figure S11 (A) shows several fungal families were more enriched in the pre-trial soil than the control. The only fungi that were enriched in the control was *Herpotrichiellaceae* and *Arthrobothrys*, the enrichment of these fungi in addition to the decrease or lack of change in the alpha diversity may explain why more species were depleted in the control versus the pre-trial soil.

The significant differences between Mustard and the Control are unclassified genera in the family *Herpotrichiellaceae* and order Hypocreales. For Ryegrass Abergain the differences are in orders Pleosporales, Hypocrales, families Herpotrichielaceae, Aspergilaceae and genera Arthrinium, and Arthrobotrys. Compared to the control, *Arthrobotrys* was enriched in the Ryegrass treatment (7-8 log2

fold change), but depleted in the mustard treatment. This fungus was identified at species level to be *Arthrobotrys oligospora,* a nematophagus fungus unlikely to be a direct influence on improving bioremediation. The family *Herpotrichiellaceae* were further enriched in the mustard compared to the control (3-4 log2 fold Change).

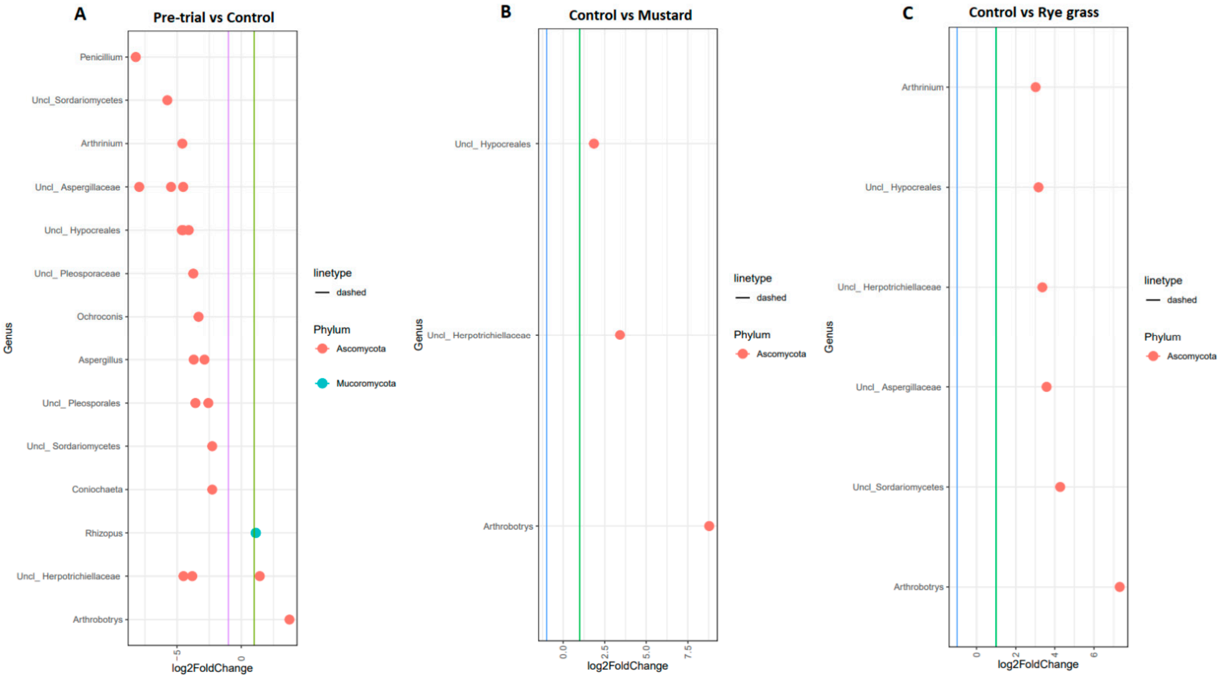

Figure S11: Differential abundance analysis of fungal ASVs. The graph shows the significant differences at genus level where possible, but family and order are also represented. Differences shown are between the control and each treatment at week 14 sampling time. Note that a differential abundance graph for Chicory was not possible as there was no significant differential abundance between the control and chicory treatments. Colours are based on phylum, the x axis measures log2 fold change.

Table S1: This table shows the layout of the randomized pot trial in the top half and a description of the plants and abbreviations used in the bottom half.

**Randomised Pot trial layout**					
M	RG1	C	Ch	RG1	RG2
RG1	C	RGWC	RGWC	RGWC	Ch
C	M	RG1	RG2	Ch	M
RG2	Ch	RG2	RG2	M	RGWC
C	M	RGWC	Ch	RG1	C
**Plant Name or variety**	**Abbreviation**	**Plant Species**	**Description of plant**	**Growth cycle**	**No. plant per pot**
Control (biostimulation)	C	N/A	N/A	N/A	0
White Mustard	M	Sinapis alba	Herbaceous plant	Annual	3
Abergain (Perennial Rye)	RG1	Lolium perenne	Grass	Perennial	20
Abergreen (Perennial Rye)	RG2	Lolium perenne	Grass	Perennial	20
Perennial Rye + White clover	RGWC	Lolium perenne/Trifolium repens	Grass/Herbaceous plant	Perennial	20
Common Chicory (Spartan)	Ch	Chicorium intybus	Herbaceous plant	Perennial	3

Table S2: Biodiversity Metrics for Bacterial Communities

Sample details	Observed ASVs	Chao 1	Simpsons Inv	Shannon	Unifrac	Weighted unfrac	Bray Curtis
**Pre-trial (week 0)**	202 ± 20	203 ± 20	17.32 ± 0.61	2.06 ± 0.04	0.396 ± 0.053	0.012 ± 0.009	0.081 ± 0.040
**Control (C) week 3**	444 ± 38	447 ± 38	8.86 ± 3.17	3.28 ± 0.0.29	0.527 ± 0.031	0.007 ± 0.004	0.245 ± 0.144
**Control (C) week 14**	790 ± 93	795 ± 94	29.64 ± 16.59	4.50 ± 0.27	0.549 ± 0.056	0.010 ± 0.005	0.284 ± 0.151
**Mustard (M) week 3**	582 ± 155	585 ± 156	20.40 ± 11.87	3.89 ± 0.60	0.576 ± 0.076	0.013 ± 0.007	0.410 ± 0.224
**Mustard (M) week 14**	816 ± 45	818 ± 45	25.82 ± 4.91	4.51 ± 0.12	0.622 ± 0.143	0.020 ± 0.020	0.337 ± 0.228
**Ryegrass 1 (RG1) week 3**	516 ± 141	522 ± 142	10.89 ± 5.12	3.31 ± 0.63	0.694 ± 0.139	0.014 ± 0.009	0.338 ± 0.185
**Ryegrass 1 (RG1) week 14**	740 ± 106	742 ± 106	25.05 ± 6.26	4.39 ± 0.23	0.564 ± 0.173	0.014 ± 0.012	0.388 ± 0.193
**Chicory week (Ch) 3**	636 ± 41	642 ± 40	14.08 ± 5.20	3.80 ± 0.22	0.406 ± 0.035	0.009 ± 0.006	0.307 ± 0.185
**Chicory week (Ch) 14**	800 ± 155	804 ± 155	26.63 ± 10.05	4.51 ± 0.35	0.512 ± 0.078	0.011 ± 0.006	0.319 ± 0.175

Table S3: Statistical significance for alpha diversity metrics for Bacteria at the different sampling times. Treatments that share the same letter are not significantly different.

Treatment	Shannon	Chao1	InvSimpson	Observed
**Pretrial**	A	A	A,B,C	A
**Control (C) Week 3**	A	B,C	A	B
**Control (C) Week 14**	B	C,D	C	C,D
**Mustard (M) week 3**	A,B	B,C,D	A,B,C	B,C,D
**Mustard (M) week14**	B	D	B,C	D
**Ryegrass 1 (RG1) week 3**	A	B,C	A,B	B,C
**Ryegrass 1 (RG1) week 14**	B	C,D	B,C	C,D
**Chicory (Ch) week 3**	A,B	B,C,D	A,B,C	B,C,D
**Chicory (Ch) week 14**	B	C,D	B,C	C,D

Table S4: Biodiversity Metrics for Fungal Communities

Sample details	**Observed ASVs**	**Chao 1**	**Simpsons Inv**	**Shannon**	**Unifract**	**Weighted unfract**	**Bray Curtis**
**Pretrial (week 0)**	78 ± 5	83 ± 5	3.85 ± 0.15	2.06 ± 0.04	0.396 ± 0.053	0.011 ± 0.008	0.081 ± 0.040
**Control (C) week 3**	73 ± 13	78 ± 11	1.97 ± 0.42	1.37 ± 0.36	0.527 ± 0.031	0.007 ± 0.004	0.245 ± 0.144
**Control (C) week 14**	46 ± 4	47 ± 4	2.37 ± 1.50	1.27 ± 0.54	0.517 ± 0.062	0.010 ± 0.005	0.286 ± 0.147
**Mustard (M) week 3**	62 ± 3	65 ± 4	2.47 ± 1.83	1.23 ± 0.56	0.576 ± 0.076	0.013 ± 0.007	0.410 ± 0.224
**Mustard (M) week 14**	49 ± 15	50 ± 16	2.41 ± 0.14	1.49 ± 0.22	0.622 ± 0.143	0.020 ± 0.020	0.337 ± 0.228
**Ryegrass 1 (RG1) week 3**	77 ± 14	79 ± 14	3.23 ± 1.13	1.80 ± 0.31	0.694 ± 0.139	0.014 ± 0.009	0.338 ± 0.204
**Ryegrass 1 (RG1) week 14**	51 ± 4	51 ± 5	4.39 ± 1.83	2.07 ± 0.37	0.564 ± 0.173	0.014 ± 0.012	0.338 ± 0.193
**Chicory (Ch) week 3**	70 ± 4	72 ± 4	2.88 ± 0.62	1.75 ± 0.26	0.406 ± 0.035	0.009 ± 0.006	0.307 ± 0.185
**Chicory (Ch) week 14**	53 ± 12	54 ± 12	3.81 ± 1.04	1.89 ± 0.31	0.512 ± 0.078	0.011 ± 0.006	0.319 ± 0.175

Table S5: This table shows the average aerial, root and total dry biomass, in addition to their standard deviations at the end of the trial.

Treatment	**Aerial biomass**	**Root biomass**	**Total biomass**
**White Mustard (M)**	8.42 ± 4.80	1.68 ± 0.67	10.1 ± 5.05
**Abergain (RG1)**	11.56 ± 1.01	5.06 ± 2.40	16.62 ± 3.04
**Abergreen (RG2)**	11.68 ± 1.30	5.82 ± 2.40	17.5 ± 3.38
**Perennial Rye + White clover (RGWC)**	9.40 ± 0.63	3.80 ± 0.94	13.2 ± 1.20
**Common Chicory (Spartan) (Ch)**	6.18 ± 0.40	5.78 ± 1.75	11.96 ± 2.00
